# The reliability of patient blood pressure self-assessments – a cross-sectional study

**DOI:** 10.1186/s12875-022-01962-x

**Published:** 2023-01-03

**Authors:** Katarzyna Nessler, Anna Krztoń-Królewiecka, Anna Suska, Mitchell R. Mann, Michał B. Nessler, Adam Windak

**Affiliations:** 1 Department of Family Medicine, Jagiellonian University Medical College, Bocheńska 4, 31-061 Kraków, Poland; 2 Department of Family Medicine, Students’ Family Medicine Interest Group, Jagiellonian University Medical College, Kraków, Poland; 3 Burns and Plastic Surgery Centre of Malopolska, Rydygier Memorial Hospital, Os. Zlotej Jesieni 1, 31-826 Kraków, PL Poland

**Keywords:** Self measurement of blood pressure, Hypertension

## Abstract

**Objective:**

Home blood pressure monitoring (HBPM) is an increasingly important tool in managing hypertension (HTN); however, its efficacy depends on its accuracy. This study aimed to explore the differences between blood pressure (BP) measurements conducted by patients and medical professionals and the patient demographic factors correlating with inaccurate self-measured BP levels.

**Methods:**

One hundred hypertensive patients completed a questionnaire inquiring about their health status and HBPM procedures and were filmed while measuring their BP using their own devices. A researcher then measured the patients' BP using a calibrated sphygmomanometer to assess the accuracy of patient-performed readings. This cross-sectional study was conducted in five primary healthcare centers in Kraków, Poland.

**Results:**

The mean differences in systolic and diastolic BP readings by patients and researchers were 8.36 mmHg (SD = 10.90 mmHg) and 2.16 mmHg (SD = 9.12 mmHg), respectively. Inaccuracies in patient BP measurements were associated with a less than high school education level, patients’ age, and a family history of HTN.

**Conclusion:**

Patient self-measured BP levels were higher than researcher values, likely due to a higher patient error rate. Healthcare providers must increase training regarding correct HBPM techniques offered to patients; such efforts should be directed at all hypertensive patients, emphasizing the most error-prone demographics.

## Key points

Most hypertensive patients make multiple errors during blood pressure self-assessments. This leads to significant discrepancies compared to readings performed by healthcare professionals.

1. Patients with hypertension must be educated regarding correct BP self-measurement practices.
2. The ability to conduct independent assessments by patients must be verified before any clinical decisions are made.

## Introduction

Hypertension (HTN), defined as a systolic blood pressure (SBP) ≥ 140 mmHg and a diastolic blood pressure (DBP) ≥ 90 mmHg, affects an estimated 1.13 billion people globally [1]; its complications kill an estimated 9.4 million people annually [2].

The increasing prevalence of HTN and greater access to BP monitors have led healthcare systems to encourage patient-conducted home BP monitoring (HBPM). HBPM is the average of BP readings performed with a semiautomatic BP monitor for at least three and preferably six to seven consecutive days. Readings should be performed in the mornings and evenings in a quiet environment following a five-minute rest period while seated with back and arm support [3]. The observations showed that out-of-office BP measurements (including home BP measurements and 24-h ABPM) present more accurately patients’ BP when compared to office measurements. These two BP measurement methods were recommended by the ESH/ESC guidelines [4]. However, the subjects’ HBPM can help in diagnosing and control of their BP only if the measurements are performed in a proper way and the data derived from BP diaries are valid.

HBPM allows for more frequent, consistent, and convenient readings while reducing strain on healthcare systems [5, 6], and has led to BP reductions amongst hypertensive patients [7–10]. Several organizations and researchers recommend HBPM for its accuracy over extended periods and potential to increase patient compliance in BP control while reducing required pharmacotherapy [11–18]. It is clear that properly performed home BP readings by well-educated patients could help doctors in everyday practice.

Despite several published detailed summaries and position papers regarding the correct methods of BP, a significant number of patients still make mistakes during their home measurements [19, 20].

Measurement inaccuracies diminish the advantages of HBPM; HBPM devices are often operated erroneously, primarily due to a lack of patient training by healthcare providers on their correct use [21–26]. HBPM can also be problematic for patients with physical handicaps or those suffering from mental decline or impaired cognition [21]. Additionally, HBPM may cause patient anxiety and stress, leading to obsessive measurements and skewed results [27]. Ultimately, HBPM inaccuracies due to patient errors negatively influence treatment decisions, leading to inappropriate prescriptions and maligned outcomes.

Previously, we assessed the common errors patients made during HBPM [28]. We determined that only 29% and 5% of patients received information regarding correct HBPM techniques from a physician or nurse, respectively; 22% of patients received no guidance [28].

In this study, we aimed to answer the following:

1. How accurate are patient BP self-measurements compared to those performed by clinicians?
2. Are there associations between patient characteristics and differences in BP measurements recorded by patients and clinicians?

## Methods

### Study design

This cross-sectional study was conducted between July 2016 and May 2018. Participants were recruited from five primary healthcare centers in Kraków, Poland. Medical students from Jagiellonian University Medical College served as fieldworkers; all researchers received instructions regarding the study protocol before the commencement of fieldwork.

Study participants signed an informed consent form and completed a demographic and clinical data questionnaire. Afterward, for five minutes they sat in a quiet room, which was unattended by any healthcare worker, and then, they independently measured their BP using their sphygmomanometers in the same manner they would at home. Patients completed two BP measurements one to two minutes apart and performed a third measurement if the first two readings differed by > 10 mmHg. BP readings were recorded as the average of the last two measurements. Patients were filmed for technique quality assessment and were aware of their surveillance.

Five minutes after the final patient-conducted BP measurement, a researcher performed BP measurements with a calibrated upper arm automatic sphygmomanometer (OMRON M3 Automatic BP Monitor). The measurements were performed according to the guidelines: taking two readings with one to two minutes interval between readings. A third measurement was made if the first two readings differed by > 10 mmHg.

This study was approved by the Jagiellonian University Bioethics Committee (122.6120.121.2015; June 25, 2015) and was conducted according to good clinical practice rules, with secured complete patient confidentiality. A description of the study design has been published previously [28].

### Participants

Participants were required to meet the following eligibility criteria: (1) age ≥ 18 years, (2) current diagnosis of HTN, (3) declared regular HBPM, (4) informed consent, (5) lack of a history of arrhythmias, and (6) lack of comorbidities that could prevent communication with investigators or bias the results (e.g., cognitive, visual, or hearing impairments, motor difficulties, inabilities to give informed consent). No restrictions were enacted to select for patients' level of HBPM training. The purpose and methods of the investigation were explained to all participants.

The minimum patient sample size (*n*) calculated with OpenEpi software was estimated to be 97. In total, 147 hypertensive patients were invited to participate in the study.

### Measurements

BP measurements were expressed in mmHg with an accuracy of ± 2 mm.

Questionnaire data included patient age, gender, education (levels 1–8 according to the European Qualifications Framework, EQF), residence (village/town < 50,000 inhabitants, city > 50,000 inhabitants), family history of HTN (positive/negative), chronic comorbidities (coronary heart disease, heart failure, diabetes mellitus type II, renal failure) and type of a HBPM sphygmomanometer used (aneroid, upper arm automatic, upper arm semiautomatic, wrist).

Patient errors were classified in our previous study with the same participants [28].

### Statistical analysis

To illustrate respondent characteristics and BP measurement values, we calculated descriptive statistics as distributions for qualitative data and means, medians, and ranges for quantitative data. The dependent *t*-test was used to analyze the differences in SBP and DBP readings between those performed by patients and researchers. Using forward stepwise multivariate regression, we assessed the associations of patient sociodemographic characteristics, sphygmomanometer type, and errors made by patients during BP self-measurements with differences in BP levels recorded by patients and researchers. An α level of *p* = 0.05 was accepted as statistically significant. Statistica 13.3 software (TIBCO Inc.) was used for all statistical analyses.

## Results

### Respondent characteristics and their BP recording errors

One hundred of the 147 invited hypertensive patients, who agreed to participate were recruited in the order in which they made a medical appointment for any reason (response rate: 68%). Detailed characteristics are presented in Table 1. Types of errors made by patients are presented in Table 2.

**Table 1:** Respondent demographic characteristics

Gender	
Female	61%
Male	39%
Age	
Mean	66.19 years (SD = 10.07 years)
Minimum	36 years
Maximum	85 years
Time from HTN diagnosis	
Mean	12.5 years (SD = 8.24 years)
Minimum	1 year
Maximum	32 years
BMI	
Mean	29.95 kg/m^2^ (SD = 4.76 kg/m^2^)
Minimum	19.37 kg/m^2^
Maximum	42.25 kg/m^2^
Education level	
Less than high school (1^st^-3^rd^ EQF level)	41%
High school equivalent (4^th^-6^th^ EQF level)	34%
University (7^th^-8^th^ EQF level)	25%
Place of residence	
Village or town with less than 50 000 inhabitants	31%
City with more than 50 000 inhabitants	69%
Family history of HTN	
Positive	63%
Negative	37%
Chronic comorbidities	
Yes	29%
No	71%
Type of sphygmomanometer	
Aneroid	11%
Upper arm automatic	64%
Upper arm semi-automatic	7%
Wrist	18%
Number of errors made by patients	
Median	3 (Q1 = 2, Q3 = 4)
Minimum	0
Maximum	6
Types of errors made by patients	
Incorrect pressure gauge cuff placement	76%
Lack of back support	70%
Incorrect upper limb placement	56%
Incorrect cuff fastening	27%
Compression of clothing on the frame	22%
Crossed legs	20%
Fingers not laid loosely	14%
Conversation during measurements	8%

**Table 2:** Types of errors made by patients

**Gender**		**Family history of HTN**	
Female	61%	Positive	63%
Male	39%	Negative	37%
**Age**		**Chronic comorbidities**	
Mean	66.19 years (SD = 10.07 years)	Yes	29%
Minimum	36 years	No	71%
Maximum	85 years	**Type of sphygmomanometer**	
**Time from HTN diagnosis**		Aneroid	11%
Mean	12.5 years (SD = 8.24 years)	Upper arm automatic	64%
Minimum	1 year	Upper arm semiautomatic	7%
Maximum	32 years	Wrist	18%
**BMI**		**Number of errors made by patients**	
Mean	29.95 kg/m^2^ (SD = 4.76 kg/m^2^)	Median	3 (Q1 = 2, Q3 = 4)
Minimum	19.37 kg/m^2^	Minimum	0
Maximum	42.25 kg/m^2^	Maximum	6
**Education level**		**Types of errors made by patients**	
Less than high school (1^st^-3^rd^ EQF level)	41%	Incorrect cuff placement	76%
High school equivalent (4^th^-6^th^ EQF level)	34%	Lack of back support	70%
University (7^th^-8^th^ EQF level)	25%	Incorrect upper limb placement	56%
**Place of residence**		Incorrect cuff fastening	27%
Village/town ˂50 000 inhabitants	31%	Compression of clothing on the frame	22%
City ˃50 000 inhabitants	69%	Crossed legs	20%
		Fingers not laid loosely	14%
		Conversation during measurements	8%

### Comparison of patient and researcher BP measurements

**Fig. 1:**
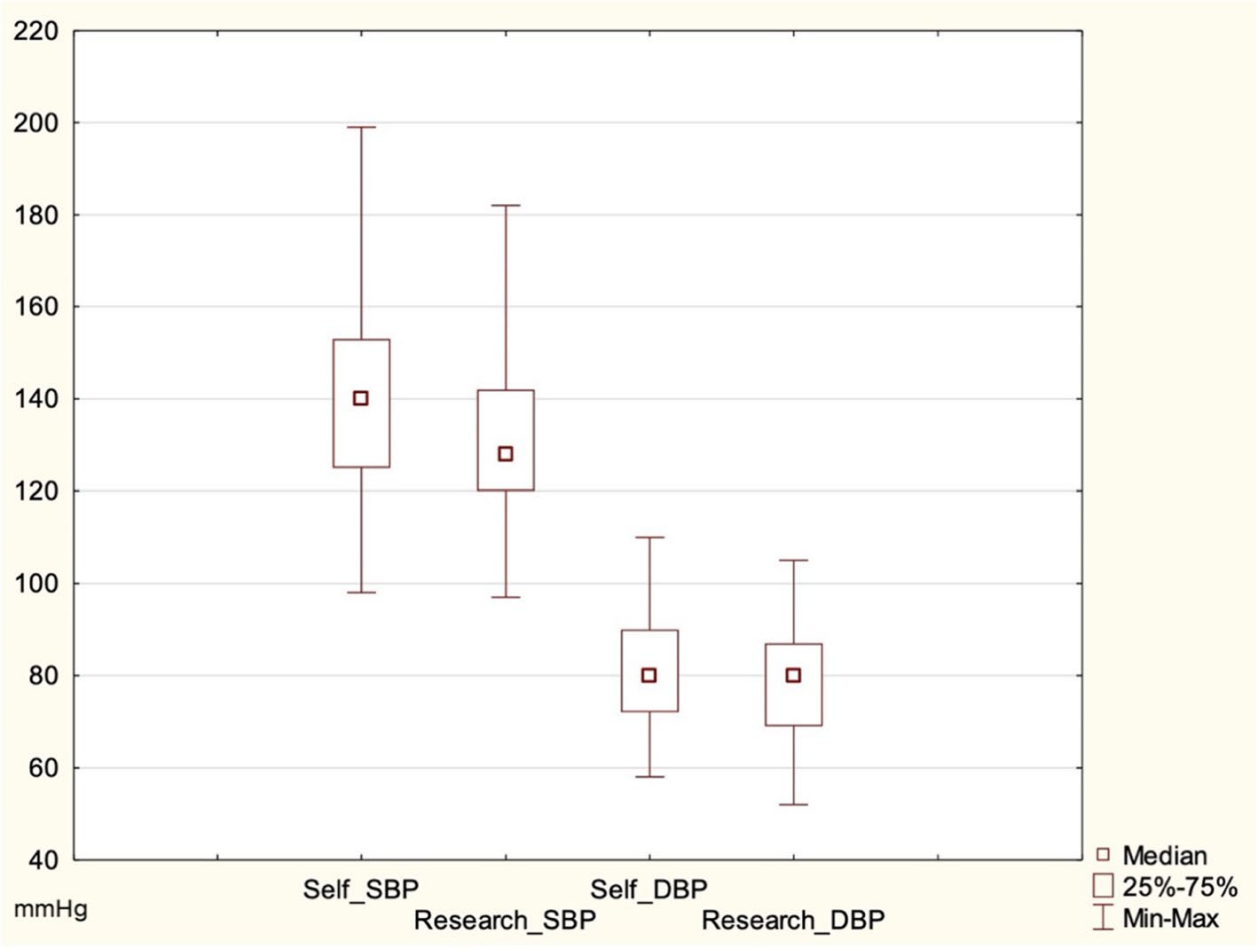
The ranges of patient and researcher SBP and DBP readings

We observed significant differences in the mean values of SBP and DBP measurements performed by patients compared to those conducted by researchers. Mean SBPs measured by patients and researchers were 140.83 mmHg (SD = 19.33 mmHg) and 132.28 mmHg (SD = 16.97 mmHg), respectively (*p* < 0.001) (Fig. 1). Mean DBP readings performed by the patients were significantly higher than those taken by researchers: 80.94 mmHg (SD = 11.76 mmHg) versus 78.76 mmHg (SD = 11.46 mmHg) (*p* = 0.020) (Fig. 1).

**Fig. 2:**
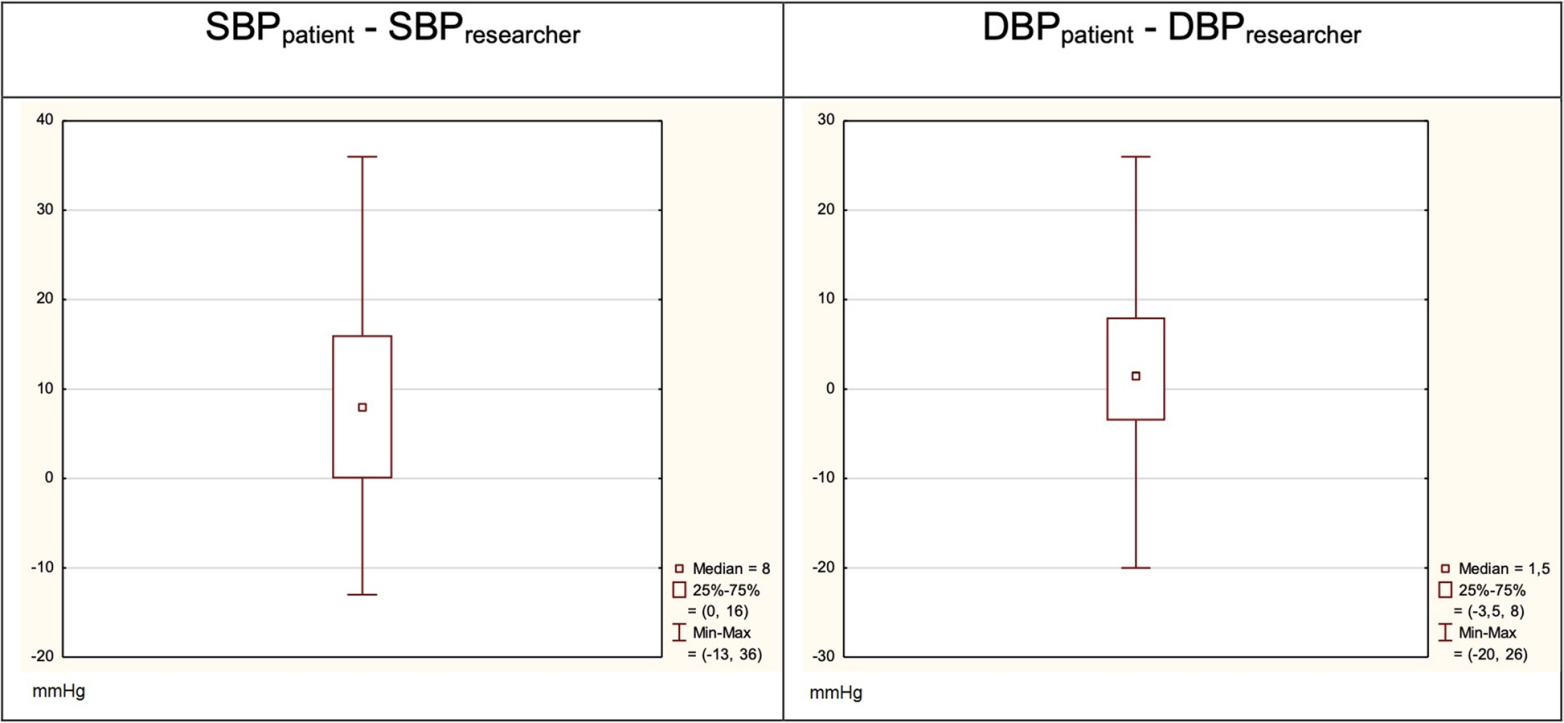
The ranges of differences in SBP and DBP measurements between patients and researchers

The mean differences in SBP and DBP readings between patients and researchers were 8.36 mmHg (SD = 10.90 mmHg) and 2.16 mmHg (SD = 9.12 mmHg), respectively (Fig. 2).

### Differences in SBP readings between patients and researchers

A lesser difference in SBP readings performed by patients and researchers was observed among patients with a high school education (4^th^-6^th^ EQF levels) compared to those with less than a high school education (1^st^-3^rd^ EQF levels) (*p* = 0.004) and patients with chronic comorbidities (*p* = 0.002) (Table 3).

**Table 3:** Stepwise forward regression model: difference in SBP mercury readings performed by patients and researchers with patient characteristics (reference group indicated in italics)

Patient characteristics				
Variable	Comparison	Beta	b	*p*
**Education**				
***Less than high school (EQF 1–3)***	**High school equivalent (EQF 4–6)**	**-0.298**	**-7.956**	**0.004**
**Chronic comorbidities**				
***No***	**Yes**	**-0.321**	**-8.899**	**0.002**
**Mistake during BP measurement: incorrect pressure gauge cuff placement**				
***No***	**Yes**	**0.259**	**3.069**	**0.011**
BMI		0.155	0.420	0.125
Mistake during BP measurement: compression of clothing on the frame				
*No*	Yes	-0.139	-4.286	0.166

### Differences in DBP readings between patients and researchers

Differences in DBP readings observed among patients with a high school education (4^th^-6^th^ EQF levels) were less pronounced than patients with less than a high school education (1^st^-3^rd^ EQF levels) (*p* < 0.001). The difference between patient and researcher readings was higher for patients with a positive family history of HTN (*p* = 0.024) and older patients (*p* = 0.040) (Table 4).

**Table 4:** Stepwise forward regression model: difference in DBP mercury readings made by patients and researchers with patient characteristics (reference group indicated in italics)

Patient characteristics				
Variable	Comparison	Beta	b	*p*
**Education**				
***less than high school (EQF 1–3)***	**high school equivalent (EQF 4–6)**	**-0.392**	**-8.192**	** < 0.001**
**Family history of hypertnsion**				
***Negative***	**Positive**	**0.241**	**4.972**	**0.024**
Mistake during BP measurement: not being in seated position				
*No*	Yes	0.116	10.520	0.275
**Age**		**0.227**	**0.230**	**0.040**
Mistake during BP measurement: no back support				
*No*	Yes	0.154	3.460	0.131
Chronic comorbidities				
*No*	Yes	-0.145	-3.148	0.169
Mistake during measurement: incorrect pressure gauge cuff placement				
*No*	Yes	-0.131	-3.166	0.199

## Discussion

### Summary of main findings

Significant differences were observed in the mean BP readings recorded by patients and investigators; SBP and DBP readings were higher when measured by patients. A high school education, compared to lower education level, was a negative predictor for the difference in both SBP and DBP readings taken by patients and researchers. Chronic comorbidities were an additional negative predictor for SBP differences. The incorrect placement of the pressure gauge cuff, the most common patient error, was a positive predictor for SBP differences between patient- and researcher-based readings. Positive predictors for DBP differences were a positive family history of HTN and older age.

### Strengths and limitations

The principal strength of this investigation is its standardized protocol in assessing the accuracy of patient-conducted readings.

This study is limited by its scope; all participants inhabit one region of Poland. However, the patient cohort displays diversity in gender, place of residence, education level, prior medical and family medical histories, and sphygmomanometer type used. Therefore, our sample can be considered representative of the broader Polish population.

It is important to consider the stresses of the examination and their effect on the accuracy of BP measurements. Performing such self-assessments in a clinical environment outside of the comfort and routine of one's home may cause a higher error rate and a greater level of inaccuracy. Patients may have also felt more rushed to perform their self-assessments than if they were not under observation.

### Comparison with other studies

Multiple studies have highlighted the deficits in patient training regarding correct HBPM techniques. A study investigating primary care physician attitudes towards HBPM showed that while 63% of primary care doctors involved in the study encouraged HBPM, only 8% of patients were given adequate training [22]. Likewise, Wong et al*.* showed that 85% of patients using automated BP devices received no training on their correct use [29]. The combination of a detailed protocol and a lack of adequate patient education reduces the accuracy of HBPM readings [30–32]. As in our study, these investigations highlight the need to improve patient education regarding correct HBPM techniques.

In a study like ours, Stryker et al. assessed the accuracy of automatic digital BP monitors and their patient users and the effects of correcting technique errors with a HBPM education program [33]. Eighty subjects owning an automated digital BP monitor recorded their BP in a clinic while supervised by an investigator who documented and corrected technique errors. Next, BP values were recorded by both the investigator and the subject simultaneously on opposite arms, and then the arms were switched. The subjects then recorded their BP a final time. Prior to technique corrections, patient self-measured BP levels were greater than those recorded by healthcare professionals, with SBP and DBP levels being 5.8 and 1.3 mmHg greater than the average of all the readings, respectively. These results were like ours, with our observed mean differences in SBP and DBP readings between patients and researchers being 9.15 mmHg (SD = 12.95 mmHg) and 2.60 mmHg (SD = 10.03 mmHg), respectively. As in our study, the authors attributed discrepancies between patient and researcher measurements to a high patient error rate. When patient techniques were corrected, the discrepancy was significantly reduced. It is foreseeable that the errors made by our patients had a similar effect on self-measured BP levels; patient education should decrease these differences.

Bancej et al*.* assessed HBPM amongst hypertensive Canadians, with inquiries regarding their HBPM practices, sociodemographic traits, and BP control [23]. It was found that 45.9% of participants regularly performed HBPM, while 29.7% received operational instructions from a healthcare provider, and 35.9% shared their readings with healthcare professionals. However, only 15.8% of subjects claimed to meet all three of these criteria. The authors arrived at a similar conclusion to our own: an inadequate amount of correct HBPM is being conducted amongst hypertensive adults and that further knowledge translation is needed to improve HBPM efficacy.

In a cluster randomized control trial, Fung et al. assessed whether a HBPM education program could improve patient BP levels [24]. The authors monitored two 120-patient groups; one participated in a HBPM education program explaining proper techniques, while the second received standard treatment without additional instructions. After three months, SBP and DBP dropped in the intervention group by 1.88 (*p* = 0.372) and 3.84 (*p* = 0.004) mmHg, respectively. However, while SBP and DBP maintained a decreasing trend, no significant decrease between the intervention and control groups was observed by six months. The authors concluded that the education program improved the outcomes of HBPM in the short term and that additional components to the program may prolong such benefits. Going off this investigation, it would be interesting to re-evaluate the same patients assessed in our study to determine if the accuracy of their self-BP measurements improved due to technique corrections.

In our study, the observed patients’ self-measurement aimed to imitate the patients’ home-measuring behavior, similar to an unattended automated measurement that was used in the SPRINT trial [34]. Our results are consistent with the SPRINT study outcome where BP values were also higher when taken unattended compared with attended BP measurements. As the results of the SPRINT study lowering the upper level of normal blood pressure was recommended in the American Hypertension Guidelines published in 2017 [35].

### Interpretation of study findings

Discrepancies in BP values measured by patients and researchers are likely due to patient errors and organic increases in BP during the readings due to added stress. However, it should be noted that BP levels measured by clinicians may also be inflated due to WCHTN.

Patients with less than a high school education and lacking other chronic comorbidities were more likely to have inaccurate BP measurements. This may be because both uneducated patients and those with fewer existing health problems are less cognizant of their health status and the methods by which it is monitored. Accordingly, they are less likely to be aware of correct HBPM techniques and the implications of inaccurate readings. Likewise, older patients and patients with family histories of HTN were more likely to have a substantial difference in DBP measurements compared to researcher-measured values, possibly due to the long periods between their diagnoses and this investigation; more time between these two points may allow for patients to forget correct HBPM techniques.

Finally, patients suffering from chronic comorbidities were less likely to make errors while measuring their BP, possibly due to having more experience with their attending healthcare professionals and better understanding correct measurement techniques.

Our findings indicate a lack of adequate patient counseling; healthcare systems must educate hypertensive patients on correct HBPM techniques to reduce error rates and increase measurement accuracy.

### Clinical implications

The increased global incidence of HTN will raise financial and labor stresses on healthcare systems, but affordable and readily available HBPM apparatuses can mitigate these effects. Leading healthcare societies recommend HBPM to control and monitor rising levels of HTN [12, 21]; notably, it reduces the needed frequency for direct medical attention and increases the number of repeatable measurements that can be standardized for the time of day and around daily patient routines.

HBPM is only viable when patients are adequately trained to monitor their BP status in an error-free, consistent, and reproducible manner. Therefore, healthcare systems must educate patients regarding correct BP self-measurement practices and verify their ability to do so before they conduct independent assessments. These efforts must be undertaken with all patients, but emphasis should be placed on those that were the most error-prone in this investigation, chiefly elderly patients who may have been diagnosed with HTN several years before practicing their HBPM assessments and those who are of a lower educational status. In doing so, a substantial increase in HBPM accuracy will be possible, improving the health management of patients and easing stresses on global healthcare systems.

## Conclusions

Most Polish hypertensive patients make multiple errors during HBPM, skewing their BP readings and leading to significant discrepancies compared to readings performed by healthcare professionals. Errors were more frequent amongst patients with lower educational attainment, a family history of HTN, and elderly patients. Regardless of the limitations of this study's scope, this investigation outlines the quantitative effects of patient errors on HBPM readings. Healthcare professionals must educate all hypertensive patients on correct HBPM protocols, focusing on those with a lower level of education, a family history of HTN, and elderly patients with long-term diagnoses of HTN.

## Data Availability

The datasets used and/or analysed during the current study available from the corresponding author on reasonable request.

## Abbreviations

BP: Blood pressure
DBP: Diastolic blood pressure
ESH: European Society of Hypertension
ESC: European Society of Cardiology
EQF: European Qualifications Framework
HBPM: Home blood pressure monitoring
HTN: Hypertension
SBP: Systolic blood pressure
